# Event-Related Potentials and Cognitive Function in Alcoholism

**Published:** 1995

**Authors:** Bernice Porjesz, Henri Begleiter

**Affiliations:** Bernice Porjesz, Ph.D., is an assistant professor in the Department of Psychiatry, State University of New York Health Science Center, Brooklyn, New York. Henri Begleiter, M.D., Ph.D., is a professor of psychiatry and neuroscience in the Department of Psychiatry, State University of New York Health Science Center, Brooklyn, New York

**Keywords:** evoked potential, cognitive process, AOD dependence, memory, AOD impairment, sensory stimuli, AOD abstinence, biological markers

## Abstract

Subtle cognitive deficits can be studied using event-related potentials (ERP), brain waves elicited in response to sensory stimuli. ERP studies suggest that certain memory processes are impaired in alcoholics; therefore, each incoming stimulus must be evaluated anew. Some ERP anomalies occur in nonalcoholic subjects from alcoholic families, indicating that these anomalies may antecede the development of alcoholism.

Cognitive impairment in alcoholics^1^ ranges from severe memory disorders and dementia to subtle impairment of the ability to process and interpret sensory information. A majority of alcoholics may exhibit subtle cognitive impairment. For many years, these milder deficits were not well understood because the sensitive techniques required for their detection were unavailable.

Today, subtle cognitive deficits can be studied using event-related potentials (ERP), a special application of the electroencephalogram (EEG). Traditional EEG techniques use electrodes placed in standard positions on the scalp to measure the spontaneous electrical activity of the brain. Characteristic EEG patterns have been correlated with different mental and behavioral states. In contrast, ERP’s are elicited in response to sensory stimuli, such as sights or sounds. Brain electrical responses can be measured within a fraction of the second that follows exposure to a stimulus, providing an immediate record of brain activity associated with information processing.

## How ERP’s Are Obtained

The brain can generate ERP’s in response to stimuli in any sensory system, or channel. In most ERP experiments, subjects are exposed to stimuli in the visual or auditory channels (or both). Visual stimuli, such as shapes or letters, can be presented on a computer screen; auditory stimuli, such as tones or clicks, can be presented through headphones. The subject may be asked to attend to some stimuli while ignoring others. Generally the subject is asked to make a behavioral response to a certain stimulus. For example, he or she may be asked to press a button whenever the stimulus is detected. Stimuli that require a response are called targets. In some tasks, the subject must attend or respond to a specific stimulus in one sensory channel while ignoring all stimuli in another sensory channel; the sensory channel containing the target stimulus is called the relevant channel.

Scalp electrodes record the subject’s brain electrical activity during the task. The electrical signals are processed electronically and displayed as undulating lines, each line forming a series of peaks (positive waves) and valleys (negative waves). Waves, or components,^2^ are named according to their polarity—positive (P) or negative (N)—and their latency. Latency is the interval, in thousandths of a second (milliseconds [msec]) between the occurrence of a stimulus and the peak of the wave.

Early ERP components (those with latency less than 100 msec) are automatic responses to physical characteristics of the stimulus. In contrast, later components reflect the subject’s processing of the stimulus and are influenced by the psychological circumstances under which the stimulus is presented.

## What ERP’s Indicate About Cognitive Processes

Various mental operations are involved in responding to a target stimulus. For example, the subject must focus attention on the relevant sensory channel, ignore irrelevant stimuli, distinguish among similar stimuli, and then recognize the target stimulus by matching it to a template stored in the memory. These operations are correlated with electrical activity in groups of nerve cells from the sense organs themselves (i.e., eyes, ears) to the highest centers of information processing in the brain. Researchers have identified certain ERP components that appear to reflect these operations. ERP components between experimental and control subjects are compared in terms of changes in latency and differences in wave height (i.e., amplitude).

### Selective Attention: N1 and Nd

N1 (or N100) is a negative wave occurring approximately 100 msec after a stimulus. N1 consists of two components that may overlap in time—an earlier automatic component followed by an attention-related component. The automatic component of N1 is the same in both the relevant and irrelevant channels. In normal subjects, N1 has a greater amplitude when elicited by stimuli in a relevant sensory channel compared with the N1 elicited by stimuli in an irrelevant channel. Therefore, to examine the attentional process, the amplitude of the N1 from the relevant channel is subtracted from that of the irrelevant channel, yielding an Nd (negative difference) component. Nd amplitude thus reflects the subject’s allocation of attentional resources to the appropriate channel.

In one experiment (Porjesz and Begleiter 1979), alcoholics who had been abstinent for an average of 1 month were presented with sequences of random single flashes and single clicks interspersed with more rarely occurring double flashes and double clicks. For each sequence, subjects were instructed to count either the double flashes or double clicks or else to ignore all stimuli in the sequence. ERP’s were recorded only to the frequent single flashes, which were either in the relevant channel (when double flashes were counted) or the irrelevant channel (when double clicks were counted). Alcoholics maintained a low amplitude of N1 regardless of channel relevance, thereby exhibiting small or absent Nd components. These results suggest that alcoholics do not differentiate between relevant and irrelevant sensory channels.

In a similar experiment using visual stimuli, Patterson and colleagues (1987) found diminished visual Nd amplitudes in abstinent alcoholics. N1 amplitudes were reduced to both the frequent nontargets and rare targets in the relevant channel. These results suggest that alcoholics may have greater difficulty with visual, compared with auditory, selective-attention tasks.

### Evaluating the Stimulus: P3

P3 (or P300), a positive component occurring from 300 to 500 msec after a stimulus, reflects “stimulus significance” (e.g., task relevance, unpredictability, and motivational factors). To elicit P3, researchers generally use oddball tasks in which rarely occurring target stimuli (i.e., oddballs) are interspersed among frequently occurring nontarget stimuli. In normal subjects, frequently occurring nontarget stimuli elicit N1’s but not P3’s, whereas oddball stimuli elicit both N1’s and P3’s.

At least two kinds of P3 have been identified: Unattended oddball stimuli elicit P3a’s, and attended oddball stimuli in a repetitive background elicit P3b’s. Most studies have dealt with the P3b component (for review, see Regan 1989).

Porjesz and colleagues (1980) used a visual task involving geometric shapes to study P3 in alcoholics abstinent an average of 2 months. The shapes included squares, triangles, and rarely occurring irregular forms interspersed in random order. Subjects were instructed to press a button in response to the rarely occurring target stimulus; in consecutive test sequences, squares alternated with triangles as rare targets. P3’s were recorded in response to the triangle, both when it was the target and when it was a nontarget.

**Figure 1:**
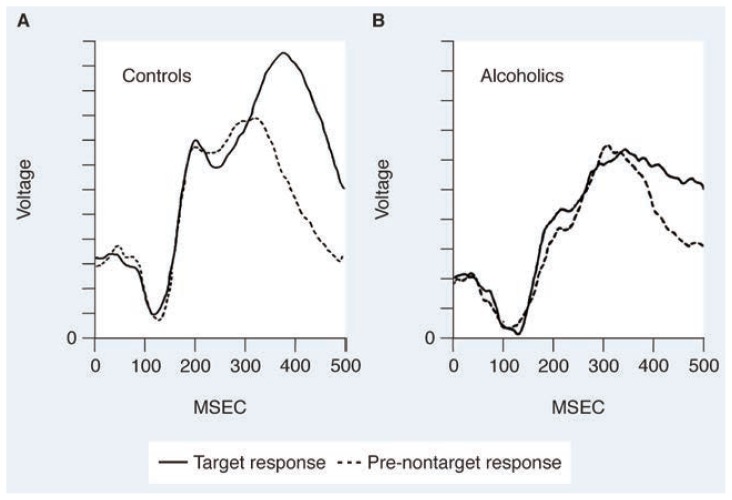
Average event-related potential (ERP) waves recorded in response to a target stimulus (solid line) and nontarget stimulus (dashed line). Target stimuli are those that require the subject to respond in some way. (A) was recorded from nonalcoholic subjects and (B) was from alcoholic subjects. Amplitude (the height of the peak) is measured in terms of the strength of the electrical signal (volts). Notice the prominent P3 component (large positive deflection occurring between 300–450 msec) in the healthy subjects. Comparison of A and B shows that this component is reduced in amplitude in alcoholic subjects. In addition, note the lack of difference between P3 amplitudes to target and nontarget stimuli in the alcoholic group.

Nonalcoholic control subjects exhibited larger late P3 components to target stimuli compared with nontarget stimuli (figure 1a). Alcoholics, however, manifested reduced or absent P3 components that differed little in amplitude, whether the stimulus was a target or a nontarget (figure 1b) (for review, see Porjesz and Begleiter 1983, 1985, and 1993).

#### Difficulty of Discrimination

Many ERP studies have demonstrated that the more deviant a rare stimulus is from the background (i.e., the more easily it can be discriminated), the larger the P3 amplitude (for review, see Regan 1989). This effect was examined in a visual oddball task using line stimuli (Porjesz et al. 1987). P3 components were obtained in response to two target lines—one that was easy to discriminate (90 degrees from a vertical nontarget line), and one that was more difficult to discriminate (only 3 degrees from the vertical nontarget).

As predicted, nonalcoholics exhibited significantly larger P3 amplitudes to the 90-degree target than to the 3-degree target. Alcoholic subjects did not exhibit significant differences in P3 amplitude between easy and difficult targets or between targets and nontargets. This indicates that alcoholic subjects find the discrimination task more difficult and are more uncertain of their responses. In addition, alcoholic subjects tend to stress speed over accuracy in some cognitive tasks. In the above study, alcoholics exhibited faster reaction times^3^ and made more errors than nonalcoholic subjects. This suggests that alcoholics may respond to stimuli without fully evaluating them (for review, see Porjesz and Begleiter 1993).

Results from P3 auditory tasks are not as consistent as those from visual P3 tasks, although several studies have reported decreased P3 amplitudes in alcoholics relative to nonalcoholics in auditory target selection and oddball tasks (Patterson et al. 1987; Porjesz et al. 1988; Pfefferbaum et al. 1991; Cohen et al. in press).

#### Stimulus Evaluation Time

Alcoholics exhibit delayed P3 latencies to attended auditory targets but not to attended visual targets. Compared with nonalcoholics, alcoholics also exhibited reaction time delays in auditory but not visual oddball tasks (Pfefferbaum et al. 1991). Auditory oddball tasks in general appear to be easier than visual tasks. Therefore, the factor influencing P3 latency delays in these studies is probably not the sensory channel involved but rather overall ease of discrimination.

In the visual line discrimination task, nonalcoholic subjects manifested significantly earlier P3 latencies to easy discriminations compared with difficult discriminations. In alcoholics, P3 latency was delayed for the easy targets, such that P3 latency was comparable for both easy and difficult targets. These results suggest that alcoholics found both discriminations difficult and adopted an undifferentiated mode of responding regardless of task requirements (for review, see Porjesz and Begleiter 1993).

Although most attention has been focused on P3b tasks in which subjects attend to target selection, a small number of studies have examined P3a. In two P3a studies using auditory oddball tasks, alcoholics manifested lower P3a amplitudes than did nonalcoholics to rare unattended stimuli (Pfefferbaum et al. 1991; Realmuto et al. 1993).

### Evaluating the Stimulus: N2

The relationship between difficulty of discrimination and speed of stimulus evaluation can be examined in visual tasks using N2, a negative component that occurs at approximately 200 msecs after a stimulus. One study (Porjesz et al. 1987) used the line target discrimination task described above, with frequently occurring vertical nontargets interspersed with both easy and difficult rare targets. Subjects were instructed to press a button as quickly as possible to all nonvertical stimuli.

In nonalcoholic subjects, the latency of N2 reflected the difficulty of discrimination, being significantly delayed for the difficult compared with the easy discriminations. In contrast, alcoholics manifested similar N2 latencies regardless of discrimination difficulty. Moreover, the N2 latency in alcoholics occurred significantly later than in nonalcoholics for both the easy and difficult discriminations, suggesting that alcoholics find both discriminations more difficult and need more time for stimulus evaluation. Other researchers have reported similar delays in N2 latency in alcoholics in visual oddball tasks (Glenn et al. 1993).

On the basis of both the N2 and P3 ERP studies, it appears that alcoholics have less efficient match-mismatch memory processes than do nonalcoholics and hence have more difficulty evaluating the potential significance of stimuli.

### Semantic Memory: N400

N400 is a negative component occurring approximately 400 to 600 msec after a stimulus. It is most typically recorded during the presentation of a sequence of contextually related words in which one of the words is semantically incongruent. For example, the sentence “I take my coffee with cream and *socks*” would elicit a large N400 to the word “socks,” whereas the sentence “I take my coffee with cream and *sugar*” would not elicit an N400 to the word “sugar.” The more incongruous the word, the larger the N400 (for review, see Kutas and Van Petten 1988).

Another way to obtain an N400 is by means of semantic priming of single words. A word is responded to more quickly and accurately if it is preceded by the same or related words (primed) than if it follows unrelated words. In the example “table—chair,” “chair” is said to be primed by “table.” Semantic priming can be measured either with reaction time or the N400 ERP. In normal subjects, unprimed words elicit larger N400’s than primed words; primed words elicit small or absent N400’s.

In a recent study examining the N400 component in alcoholics, subjects were required to indicate as rapidly as possible whether a given sequence of letters formed a word. Words preceded by their antonyms (e.g., hot-cold) were more quickly recognized as words than were those preceded by unrelated words or nonsense syllables. In nonalcoholic subjects, the N400 component was elicited by the unprimed words but not by the primed words. However, alcoholics exhibited N400’s to primed and unprimed words alike (Porjesz and Begleiter 1994). This study marks the first time semantic memory deficits have been demonstrated in alcoholics using measures of brain electrical response.

### Memory Potentials

Priming deficits are not limited to semantic stimuli. The visual memory potential (VMP), occurring about 240 msec after a stimulus, is elicited in visual matching tasks using pictures of faces or objects, or shapes. In nonalcoholics, primed or matching stimuli elicit smaller and faster VMP’s than do nonmatching stimuli. In contrast, alcoholics manifest similar VMP’s to both matching and nonmatching stimuli in terms of both amplitude and latency. These results suggest that alcoholics do not use available information about the physical features of stimuli to facilitate or prime their responses to identical stimuli (Porjesz and Begleiter 1994).

### Memory Dysfunction and Disinhibition

Taken together, the research described above suggests that alcoholics manifest two types of memory dysfunction: The low P3 amplitude suggests that the template matching processes are impaired, and the delay in N2 latency suggests that the template itself may be lost or absent. Furthermore, these findings suggest a lack of inhibition in alcoholics as reflected by their apparent inability to withhold responding until the certainty of accuracy or correctness has been established.

Evidence from monkey studies indicates there is less nerve cell electrical activity (firing) in response to repeated or primed stimuli, suggesting selective inhibition of masses of nerve cells (Miller et al. 1991). This differential inhibition facilitates the efficient processing of a familiar stimulus. This process seems to be impaired in alcoholics, such that each incoming stimulus must be evaluated anew.

## Recovery of Cognitive Functioning With Abstinence

Research indicates that alcoholics continue to manifest low P3 amplitudes after 3 to 10 years of abstinence. In contrast, abnormalities in certain early sensory components known as brainstem auditory evoked potentials (BAER) recover completely. In addition, a study conducted in Spain indicates that while P3 does not recover following 4 months of abstinence, N2 latency recovers completely (Grau et al. 1992). These observations suggest that anomalies of some components (e.g., BAER, N2) may be consequences of heavy drinking, whereas anomalies that do not recover with prolonged abstinence (e.g., P3) may antecede the development of alcoholism.

In this regard, it is noteworthy that N2 latency may predict whether a recovering alcoholic will resume drinking. Glenn and colleagues (1993) evaluated alcoholics in a visual oddball task after approximately 1 month of abstinence and again after 1 year. Subjects who resumed drinking within the year manifested longer N2 latencies at initial testing than did those who remained abstinent. Results were not affected by a subject’s family history of alcoholism. Thus, whereas P3 amplitude may predict susceptibility to developing alcoholism (see below), N2 latency may predict susceptibility to relapse (for review, see Porjesz and Begleiter 1993).

## Family History and Risk of Alcoholism

The cognitive and other brain abnormalities observed in alcoholics have traditionally been attributed to the toxic effects of alcohol, nutritional deficits, or an interaction of toxic and nutritional factors. Increasing evidence suggests that at least some ERP aberrations may antecede the development of alcoholism.

Population genetics studies suggest that genetic factors predispose sons of alcoholic fathers to alcoholism (for review, see Hesselbrock in press). Evidence indicates that characteristics of both the EEG and ERP are themselves genetically determined. For example, the heritability of P3 amplitude recently has been reported to be high for both visual (Porjesz et al. 1993) and auditory P3 (O’Connor et al. 1994).

The first study to indicate that P3 amplitude is significantly reduced in boys at risk for alcoholism was undertaken 10 years ago (Begleiter et al. 1984). Subjects at risk were sons of alcoholic fathers; age-matched control subjects had no family history of alcohol problems. All subjects were between 7 and 13 years of age and did not drink alcohol. The task involved discrimination of rarely occurring easy and difficult visual stimuli representing greatly simplified views of a head from above. Further research has replicated these findings for both postpubescent (O’Connor et al. 1986) and prepubescent (Hill and Steinhauer 1993*a*) sons of alcoholics. Similar results have been obtained with other visual tasks (Whipple et al. 1988, 1991; Noble 1990; Berman et al. 1993; Porjesz and Begleiter 1990) as well as with an auditory task (Begleiter et al. 1987).

A recent meta-analysis^4^ (Polich et al. 1994) reported a consensus that P3 amplitude is decreased in subjects at risk for alcoholism. This decrease is most likely to be observed in prepubescent males using difficult visual tasks. Results in older offspring are more variable, particularly with easy auditory tasks.

A study by Berman and colleagues (1993) suggests that P3 amplitude in prepubescent boys may predict later alcohol or other drug (AOD) abuse in adolescence. Four years after initial ERP testing, adolescents were administered a questionnaire dealing with AOD use. Subjects exhibiting the lowest P3 amplitudes at initial testing had the highest AOD use scores in adolescence. These findings provide strong evidence that P3 amplitude in prepubescent boys may serve as a vulnerability marker for the development of later AOD use disorders.^5^

The studies discussed above have used males as subjects almost exclusively. Results of studies in females have been inconsistent. A recent nationwide study with very large sample sizes found lower P3 amplitudes in female alcoholics and their first-degree relatives,^6^ although not to the same extent as in males (Porjesz et al. 1993). In both men and women, shorter P3 latencies in nonalcoholic siblings suggest a protective effect against the development of alcoholism (Hill and Steinhauer 1993*a*, *b*).

## Summary and Conclusions

Research indicates that various ERP components are aberrant in alcoholics under certain conditions. These aberrations are associated with impaired ability to match a stimulus with the memory template of a familiar stimulus. On a more basic level, the alcoholic may have difficulty retrieving the template; it remains to be determined whether the template has not been laid down or is lost.

Some aberrant components are obtained in subjects at risk for alcoholism, indicating that these ERP anomalies may antecede the development of alcoholism. The reduced P3 amplitude in high-risk subjects may serve as a marker for susceptibility to the development of alcoholism. However, a reduced P3 amplitude alone may not necessarily be specific for alcoholism. A cluster of ERP measures (P3, N400, VMP) suggests disinhibition of nerve cell firing and together may represent a more specific marker for a predisposition for alcoholism than reduced P3’s alone.

Not all individuals manifesting these potential markers will necessarily develop alcohol problems or the disease of alcoholism. Longitudinal family studies are underway as part of the Consortium on the Genetics of Alcoholism (COGA) project to examine alcoholic and nonalcoholic family members over time. It is hoped that this approach will elucidate the link between measures of risk and the development of alcoholism. As these ERP measures are genetically determined, the data imply that a predisposition or vulnerability to alcoholism is inherited. The role of the gene-environment interaction is not to be minimized in determining whether an individual manifesting this predisposition goes on to abuse alcohol.

## Footnotes

1 Throughout this article, terms such as “alcoholism,” “heavy drinking,” and “alcohol abuse” are used. These terms are not used consistently in the alcoholism literature; therefore, the wording in each case is based on the terms used in the reference cited.

2 Although the terms “wave” and “component” are often used interchangeably, a given wave may actually represent a summation of two or more components overlapping in time.

3 Reaction time is less precise than ERP latency as a measure of the speed of information processing, because the reaction time depends on a combination of several cognitive processes followed by motor response.

4 A meta-analysis is a statistical evaluation of data compiled from several studies.

5 As with P3, the cognitive components N400 and VMP are aberrant in both alcoholics and subjects at risk (Porjesz and Begleiter 1994).

6 First-degree relatives include parents, children, and siblings.
